# Sustained high HIV incidence among men who have sex with men in Jiangsu province, China: based on the limiting-antigen avidity EIA method and six consecutive surveys, 2016–2021

**DOI:** 10.3389/fpubh.2023.1277570

**Published:** 2023-11-20

**Authors:** Ying Zhou, Yuheng Chen, Jing Lu, Zhi Zhang, Qi Sun, Xiaoyan Liu, Xiaoqin Xu, Xuerong Ya, Haiyang Hu

**Affiliations:** ^1^Institute of STD/AIDS Control and Prevention, Jiangsu Provincial Center for Disease Control and Prevention, Nanjing, China; ^2^Section of STD/AIDS Control and Prevention, Suzhou Municipal Center for Disease Control and Prevention, Suzhou, China

**Keywords:** HIV, incidence, men who have sex with men (MSM), limiting-antigen avidity EIA, China

## Abstract

**Background:**

The epidemic of HIV infection among men who have sex with men (MSM) is a major public health concern in some parts of China, but data on trends in HIV incidence are limited. This study aimed to examine the trends in HIV incidence and factors associated with recent HIV infection among MSM in Jiangsu province, China, based on the limiting-antigen avidity enzyme immunoassay (LAg-Avidity-EIA) method.

**Methods:**

Six consecutive surveys were implemented among MSM throughout Jiangsu province from 2016 to 2021. Participants were recruited in three ways. Socio-demographic and behavioral information were collected through face-to-face interviews. Venous blood samples were taken to test for HIV and syphilis. HIV incidence was estimated using the LAg-Avidity-EIA method. Chi-square trend tests were used to observe trends over the years. Multivariate regression analyses were used to identify factors associated with recent HIV infection.

**Results:**

A total of 15,401 participants were enrolled in the study. The prevalence of HIV infection ranged from 8.0 to 9.8%, with no consistent rise or fall over the years (*P* = 0.189). HIV incidence ranged from 5.0 to 9.0%, and no uptrend or downtrend was shown (*P* = 0.418). MSM who lived locally for more than 2 years (aOR = 1.366, *P* = 0.019), had a lack of comprehensive HIV knowledge (aOR = 1.643, *P* = 0.031), had engaged in unprotected anal intercourse (UAI) in the past 6 months (aOR = 7.373, *P* < 0.001), had been tested for HIV within 12 months (aOR = 1.292, *P* = 0.035), and tested positive for syphilis (aOR = 2.840, *P* < 0.001) were likely to be recently infected with HIV.

**Conclusions:**

HIV incidence among MSM has remained at a high level in Jiangsu province. In China, health education, condom use, and HIV/syphilis testing should continue to be top priorities for HIV prevention among MSM to reduce HIV transmission.

## Introduction

The international community is working to achieve the Joint United Nations Programme on HIV/AIDS (UNAIDS) 95-95-95 targets to end the AIDS epidemic by 2030. Detection and treatment of cases are implemented as the most important measures, including detecting recent infections to understand the epidemic situation and evaluate the effectiveness of interventions in order to control the epidemic (1). According to the latest data from UNAIDS, an estimated 39 million people worldwide were living with HIV at the end of 2022, including an estimated 1.3 million new HIV infections in 2022 (2). China's AIDS epidemic is also concerning, with 1.053 million people living with HIV reported in the country by the end of 2020. The proportion of homosexual transmission rose from 9.1% in 2009 to 23.3% in 2020 (3).

Jiangsu province is an economically developed eastern province of China, with a population of more than 80 million. There were 38,851 people living with HIV reported in the province as of the end of October 2022, and from January to October of 2022, men who have sex with men (MSM) accounted for 53.1% of newly diagnosed infections, far higher than the national proportion (4). AIDS sentinel surveillance results showed that the prevalence of HIV infection among MSM in the province continued to maintain a high level, with approximately 8–10% from 2011 to 2015 (5). MSM had become the highest-risk group for AIDS in Jiangsu province.

Estimation of HIV incidence is critical to monitoring current epidemic dynamics, assessing prevention efforts, identifying at-risk populations, and guiding resource allocation. Traditional prospective cohort study methods for morbidity studies are limited due to their high cost, their time-consuming nature, and potential bias. Purely mathematical modeling methods have limited the scope of application. Inaccurate prevalence and mortality data can lead to deviations in study results (6). In addition, there are convenient laboratory methods for assessing HIV incidence (6), and the most commonly used methods are BED IgG-capture enzyme immunoassay (BED-CEIA, BED for short) (7) and limiting-antigen avidity enzyme immunoassay (LAg-Avidity-EIA) (8).

In order to understand HIV incidence among MSM in Jiangsu province, we used the BED method in an AIDS sentinel surveillance survey between 2011 and 2015 to estimate it, and it was found to be high, at approximately 5–7% per year (5). Some literature has suggested that HIV incidence may be overestimated because of the probability of misclassifying long-term infections as recent using the BED method (9). The estimation of HIV incidence is more accurate based on the LAg-Avidity-EIA method due to a low misclassification rate. The method itself, however, has high reproducibility and stability (10, 11). However, it is necessary to exclude unambiguous previously diagnosed cases because misclassification cannot be completely avoided. Finally, the results need to be corrected with the false recent rate (FRR). Generally, HIV incidence among high-risk groups from AIDS surveillance sentinels is calculated in a province to reduce bias (12). LAg-Avidity-EIA has been widely used in many countries, especially in developing countries, to estimate HIV incidence in high-risk populations (13–16). In this study, the LAg-Avidity-EIA method was used to detect recent HIV infections in AIDS sentinel surveillance surveys in Jiangsu province from 2016 to 2021 to observe the trends of HIV incidence and to determine the factors associated with recent HIV infection among MSM.

## Materials and methods

### Study design and participants

Consecutive cross-sectional surveys were conducted among MSM at HIV/AIDS surveillance sites in Jiangsu province from April to July 2016 to 2021. MSM were enrolled in the survey located in any of the province's eight cities (Nanjing, Wuxi, Xuzhou, Changzhou, Suzhou, Yancheng, Yangzhou, and Zhenjiang). Eligible participants were biologically male at birth, 18 years of age or older, and self-reported having had anal or oral sex with another man in the past year. Each recruited participant had a face-to-face questionnaire interview and specimen collection. All participants provided written informed consent prior to enrolment. The implementation of HIV/AIDS surveillance sites is a routine part of disease control and prevention, so this study was exempt from ethical review.

### Sampling and recruitment

Three convenient sampling methods were used to recruit participants. (1) Recruitment at MSM gathering venues: staff of local centers for disease control and prevention (CDCs) and volunteers from local MSM community-based organizations (CBOs) conducted field surveys at venues where MSM often gathered. Interested and eligible participants were referred to the interviewers by venue owners or staff (such as bars, clubs, and bathhouses). In public places where MSM often congregated (such as parks or public restrooms), volunteers from CBOs introduced interested and eligible participants to participate in the survey.

(2) Online recruitment: recruitment information including survey period, survey site, recruitment criteria, and contact phone number, etc., were posted through QQ, WeChat, and Blued by volunteers. Eligible participants went to the designated site to complete the questionnaire and sample collection.

(3) Recruitment at VCT clinics: eligible MSM who attended a VCT (HIV voluntary counseling and testing) were recruited to the study.

The survey was anonymous throughout the study, and any information that could identify individual participants was not included in the final data. Participants' mobile phone numbers were retrieved for notification of test results and referrals to HIV/syphilis infection-related services. In order to prevent duplicate participation, the same interviewer was assigned to each survey site to identify participants by facial and other features. Besides, participants were asked if they had already taken part in a similar survey, and the staff used a computer to check participants' mobile phone numbers before the interview to prevent duplicates. In our study, there was no duplication of participation between on-site, online, and VCT recruitment.

### Measures

Participants were interviewed by interviewers who had completed a provincial or municipal training course, in a face-to-face manner. Information on the questionnaire included socio-demographic characteristics (age, marital status with women, education level, registered residence, duration of living locally, and recruitment source) and HIV-related behaviors (sexual orientation, main way of finding a sexual partner, comprehensive HIV knowledge, unprotected anal intercourse (UAI) in the past 6 months, sexually transmitted infection (STI) in the past 12 months (self-reported, including syphilis, gonorrhea, genital chlamydia trachomatis infection, condyloma acuminatum, and genital herpes), and HIV testing in the past 12 months). After the interviewee had completed the questionnaire interview, the quality of the questionnaire was checked by the quality control personnel who had completed the training course. In the study, UAI was defined as inconsistent condom use with a male partner. Not having anal sex with a male partner was equivalent to using condoms all the time. Eight questions were used to assess participants' AIDS-related knowledge, with comprehensive knowledge defined as answering six or more questions correctly (17).

Venous blood samples of each participant were collected after the questionnaire interview and serological tests were used to detect HIV and syphilis. Plasma HIV antibodies were tested using an enzyme-linked immunosorbent assay (ELISA) reagent (Zhuhai Livzon Diagnostics Inc., Zhuhai, China). For participants who tested positive for HIV, the same blood sample was retested using another ELISA reagent (Beijing Wantai Biological Pharmacy Enterprise Co., Ltd., Beijing, China). If both tests were positive, CDC staff would contact the participant through his reserved mobile phone number to draw a second blood sample for confirmatory testing (WB, western blot assay, MP Biomedical Asia Pacific Pte. Ltd., Singapore). If participants could not be reached after the initial test, their previous blood samples were used for confirmatory testing. HIV infection was defined as having a positive confirmatory testing result. LAg-Avidity-EIA (HIV-1 incidence EIA reagent, Beijing Kinghawk Pharmaceutical Co., Ltd., Beijing, China) tests were conducted for all participants infected with HIV except for unambiguous previously diagnosed cases (such as AIDS cases, patients receiving antiretroviral therapy, and cases diagnosed more than 6 months ago). Screening of syphilis was performed by an ELISA reagent (Beijing Wantai), and confirmation was conducted using a toluidine red untreated serum test (TRUST, Beijing Wantai). Syphilis infection was defined as testing positive twice, and participants were referred to a designated hospital for treatment.

LAg-Avidity-EIA was performed in an HIV confirmation laboratory in Jiangsu provincial CDC according to the instructions provided by the manufacturer. The optical density (OD) of specimens was normalized to ODn by a ratio using a calibrator (ODn = specimen OD/calibrator OD) to minimize internal variations. During the screening mode, if the ODn of a specimen is >2.0, no further testing is required and the specimen is considered a long-term seroconversion. If the ODn is ≤2.0, the specimen must be subjected to confirmatory testing (test in triplicate). During confirmatory mode, if the ODn is ≤1.5, the specimen was considered a recent seroconversion. If the ODn is >1.5, the specimen was considered a long-term seroconversion.

### Statistical analysis

The estimated HIV incidence was calculated using Microsoft Excel. Annual HIV incidence was calculated using the following consensus formula (12):


$$\begin{array}{l} I=\frac{R-FRR\times P^{\prime }}{\left(1-FRR\right)\times \left(w/365\right)\times N^{\prime }}\times 100\% \end{array}$$

The 95% confidence interval (CI) for the incidence estimate is


$$\begin{array}{l} 95\%\;CI=I\pm 1.96\frac{I}{\sqrt{\;}R} \end{array}$$

Here, *I* was the incidence; *R* was the number of recent HIV infections; *N* was the number of HIV-negative participants; *P* was the number of requested LAg-Avidity-EIA tests; *P*′ was the number of actual LAg-Avidity-EIA tests performed; *N*′ was the number of HIV-negative participants after adjustment, *N*′ = *N*×(*P*′/*P*). *w* was 130 days, which was the window period of LAg-Avidity-EIA (the longest time from seroconversion to recent infection that could be judged by avidity assay) and *FRR* was 2.3%, which was the probability of misclassifying long-term infections as recent infections by avidity assay. These two parameters were obtained from Chinese CDC (18).

Questionnaire data were double-entered and checked for accuracy at each survey site using EpiData software (version 3.1). According to the data distribution, the qualitative variables were merged and the quantitative variables were grouped. Socio-demographic and HIV-related behavioral characteristics of participants were descriptively analyzed using frequency. Chi-square tests and trend tests were used to compare differences between years and observe trends over time. Factors associated with recent HIV infection were first assessed using univariate logistic regression analysis. Variables with *P* < 0.20 were entered into a multivariable logistic regression model to identify independent factors. Multivariable analysis was conducted using a forward LR method in order to determine the adjusted odds ratios (aORs). All analyses were performed using SPSS software (version 19.0). *P* < 0.05 was considered statistically significant.

## Results

### Study population

**Figure 1:**
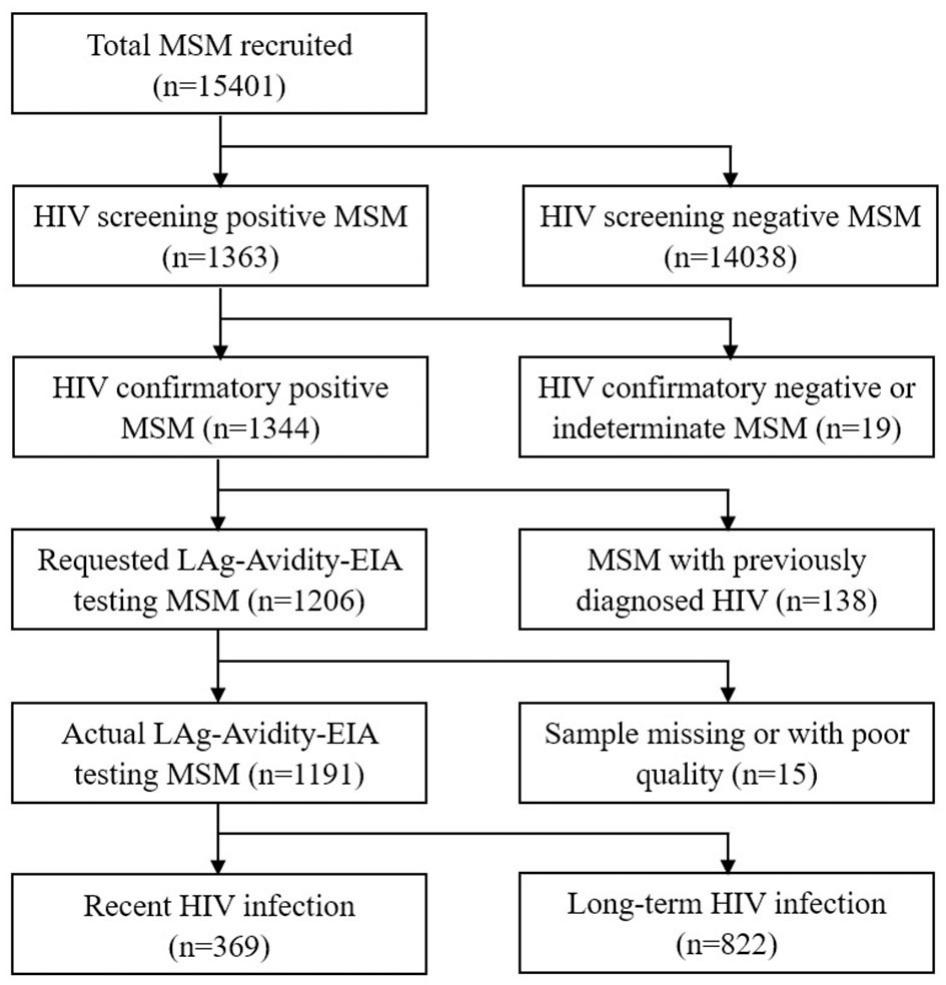
Study flowchart of MSM in sentinel surveillance surveys in Jiangsu province, China, 2016–2021.

A total of 15,401 eligible MSM (2,404, 2,656, 2,604, 2,469, 2,611, and 2,657 in 2016, 2017, 2018, 2019, 2020, and 2021, respectively) were enrolled in the study. Overall, 1,363 MSM were screened as HIV-positive, among whom 1,344 were confirmed positive, 14 tested indeterminate, and 5 tested negative using Western Blot. Of the 1,344 confirmed positive MSM, 138 (10.3%) were previously diagnosed cases, i.e., more than 6 months ago, leaving 1,206 (89.7%) newly diagnosed cases or cases diagnosed within 6 months. Of the 1,206 cases, 15, whose samples were missing or of poor quality, were not tested for LAg-Avidity-EIA testing. Of 1,191 cases receiving LAg-Avidity-EIA testing, 369 (31.0%) had recent HIV infection and 822 (69.0%) had long-term HIV infection (Figure 1).

### Trends in socio-demographic characteristics and HIV-related behaviors

In the 6-year period, as time went on, MSM who were married or cohabiting (*P* = 0.042), had a college education or above (*P* = 0.026), were part of a migrant population (*P* < 0.001), and found sexual partners via the Internet (*P* < 0.001) were more likely to participate in the surveys. The proportion of MSM recruited via the Internet increased from 20.7% in 2016 to 33.4% in 2021 (*P* < 0.001). Rates of comprehensive HIV knowledge increased from 91.8% in 2016 to 95.1% in 2021 (*P* < 0.001). Rates of UAI in the past 6 months decreased from 34.2% in 2016 to 14.6% in 2021 (*P* < 0.001). Rates of HIV testing in the past 12 months showed a significant decrease, from 56.0% in 2016 to 48.7% in 2021 (*P* < 0.001) (Table 1).

**Table 1:** Trends in socio-demographic characteristics, HIV-related behaviors, and the prevalence of syphilis and HIV infection of MSM in sentinel surveillance surveys in Jiangsu province, China, 2016-2021.

**Variable**	**2016**	**2017**	**2018**	**2019**	**2020**	**2021**	**Trend test, P-value**
	**(*****N** =* **2,404)**	**(*****N** =* **2,656)**	**(*****N** =* **2,604)**	**(*****N** =* **2,469)**	**(*****N** =* **2,611)**	**(*****N** =* **2,657)**	
	**N (%)**	**N (%)**	**N (%)**	**N (%)**	**N (%)**	**N (%)**	
**Age group (years)**							0.660
< 25	697 (29.0)	843 (31.7)	764 (29.3)	837 (33.9)	874 (33.5)	820 (30.8)	
25–39	1,179 (49.0)	1,223 (46.1)	1,211 (46.5)	950 (38.5)	1,072 (41.0)	1,195 (45.0)	
≥40	528 (22.0)	590 (22.2)	629 (24.2)	682 (27.6)	665 (25.5)	642 (24.2)	
**Marital status with women**							0.042
Married or cohabiting	731 (30.4)	812 (30.6)	828 (31.8)	812 (32.9)	858 (32.9)	850 (32.0)	
Single, divorced, or widowed	1,673 (69.6)	1,844 (69.4)	1,776 (68.2)	1,657 (67.1)	1,753 (67.1)	1,807 (68.0)	
**Education level**							0.026
Junior high school or below	627 (26.1)	687 (25.9)	774 (29.7)	622 (25.2)	658 (25.2)	700 (26.4)	
Senior high school	816 (33.9)	950 (35.8)	818 (31.4)	843 (34.1)	908 (34.8)	806 (30.3)	
College or above	961 (40.0)	1,019 (38.3)	1,012 (38.9)	1,004 (40.7)	1,045 (40.0)	1,151 (43.3)	
**Registered residence**							< 0.001
Jiangsu province	1,730 (72.0)	1,980 (74.5)	1,832 (70.4)	1,693 (68.6)	1,744 (66.8)	1,819 (68.5)	
Other provinces	674 (28.0)	676 (25.5)	772 (29.6)	776 (31.4)	867 (33.2)	838 (31.5)	
**Duration of living locally**							0.010
< 2 years	892 (37.1)	800 (30.1)	951 (36.5)	797 (32.3)	801 (30.7)	886 (33.3)	
≥2 years	1,512 (62.9)	1,856 (69.9)	1,653 (63.5)	1,672 (67.7)	1,810 (69.3)	1,771 (66.7)	
**Recruitment source**							< 0.001
Venue	1,254 (52.2)	1,288 (48.5)	1,218 (46.8)	1,004 (40.6)	869 (33.3)	816 (30.7)	
Online	498 (20.7)	639 (24.1)	720 (27.6)	772 (31.3)	922 (35.3)	886 (33.4)	
VCT clinic	652 (27.1)	729 (27.4)	666 (25.6)	693 (28.1)	820 (31.4)	955 (35.9)	
**Sexual orientation**							0.002
Bisexual or indetermination	917 (38.1)	868 (32.7)	992 (38.1)	1,046 (42.4)	947 (36.3)	936 (35.2)	
Homosexual	1,459 (60.7)	1,517 (57.1)	1,304 (50.1)	1,241 (50.3)	1,386 (53.1)	1,417 (53.3)	
Heterosexual	28 (1.2)	271 (10.2)	308 (11.8)	182 (7.4)	278 (10.6)	304 (11.4)	
**Main way of finding a sexual partner**							< 0.001
MSM venue	1,071 (44.6)	1,122 (42.2)	1,103 (42.4)	941 (38.1)	895 (34.3)	646 (24.3)	
Internet or dating app	1,333 (55.4)	1,534 (57.8)	1,501 (57.6)	1,528 (61.9)	1,716 (65.7)	2,011 (75.7)	
Comprehensive HIV knowledge	2,207 (91.8)	2,473 (93.1)	2,370 (91.0)	2,362 (95.7)	2,504 (95.9)	2,526 (95.1)	< 0.001
UAI in the past 6 months	822 (34.2)	1,000 (37.7)	835 (32.1)	658 (26.7)	519 (19.9)	387 (14.6)	< 0.001
STI in the past 12 months	142 (5.9)	183 (6.9)	136 (5.2)	119 (4.8)	119 (4.6)	148 (5.6)	0.015
HIV testing in the past 12 months	1,346 (56.0)	1,584 (59.6)	1,468 (56.4)	1,250 (50.6)	1,313 (50.3)	1,293 (48.7)	< 0.001
Syphilis infection	185 (7.7)	192 (7.2)	146 (5.6)	171 (6.9)	131 (5.0)	178 (6.7)	0.014
HIV infection	231 (9.6)	217 (8.2)	224 (8.6)	241 (9.8)	218 (8.3)	213 (8.0)	0.189

VCT, HIV voluntary counseling and testing; UAI, Unprotected anal intercourse; STI, Sexually transmitted infection.

### Trends in HIV and syphilis prevalence and HIV incidence

In the 6-year period, no uptrend or downtrend of HIV incidence was shown (*P* = 0.418), with 7.4% (95% CI: 5.5–9.2%) in 2016, 5.0% (95% CI: 3.6–6.5%) in 2017, 7.9% (95% CI: 6.1–9.8%) in 2018, 6.9% (95% CI: 5.1%-8.6%) in 2019, 9.0% (95% CI: 7.0–11.0%) in 2020, and 6.3% (95% CI: 4.7–8.0%) in 2021, respectively (Table 2). Furthermore, 231 (9.6%), 217 (8.2%), 224 (8.6%), 241 (9.8%), 218 (8.3%), and 213 (8.0%) participants tested HIV positive from 2016 to 2021, respectively. The prevalence of HIV infection ranged from 8.0 to 9.8%, but there was no consistent rise or fall over the years (*P* = 0.189). In contrast, a significant decrease was shown in the prevalence of syphilis infection (*P* = 0.014) (Table 1).

**Table 2:** HIV incidence among MSM in sentinel surveillance surveys in Jiangsu province, China, 2016–2021.

**Year**	**Negative (N)**	**Adjusted negative (N^′^)**	**Requested LAg-avidity-EIA testing (P)**	**Actual LAg-avidity-EIA testing (P^′^)**	**Recent (R)**	**Incidence (%) (95% CI) (I)**
2016	2,172	2,143	223	220	60	7.4 (5.5–9.2)
2017	2,435	2,375	204	199	46	5.0 (3.6–6.5)
2018	2,379	2,379	196	196	70	7.9 (6.1–9.8)
2019	2,226	2,160	202	196	56	6.9 (5.1–8.6)
2020	2,387	2,387	192	192	79	9.0 (7.0–11.0)
2021	2,444	2,431	189	188	58	6.3 (4.7–8.0)

The number of unambiguous previously diagnosed cases was 8, 13, 28, 39, 26, and 24, respectively, in 2016, 2017, 2018, 2019, 2020, and 2021; The number of Western Blot indeterminate samples was 1, 4, 1, 2, 6, and 0, respectively; The reasons for not testing LAg-Avidity-EIA (difference between P and P′) were samples missing or poor quality.

### Factors associated with recent HIV infection

The potential factors associated with recent HIV infection were analyzed using a univariate logistic regression model with HIV-negative MSM in 2021 as a control because some of the MSM involved were recruited more than once over the years. In the univariate logistic regression analysis, the results showed that recent HIV infection was significantly associated with duration of living locally, recruitment source, sexual orientation, comprehensive HIV knowledge, UAI in the past 6 months, STI in the past 12 months, and syphilis infection (all *P* < 0.05).

In the multivariate logistic regression analysis, MSM who lived locally for more than 2 years (aOR = 1.366, 95% CI: 1.054–1.770, *P* = 0.019), had a lack of comprehensive HIV knowledge (aOR = 1.643, 95% CI: 1.046–2.580, *P* = 0.031), had engaged in UAI in the past 6 months (aOR = 7.373, 95% CI: 5.795–9.380, *P* < 0.001), had been tested for HIV within 12 months (aOR = 1.292, 95% CI: 1.018–1.639, *P* = 0.035), and tested positive for syphilis (aOR = 2.840, 95% CI: 1.972–4.091, *P* < 0.001) were likely to be recently infected with HIV (Table 3).

**Table 3:** Univariate and multivariate logistic regression analysis of factors associated with recent HIV infection among MSM in sentinel surveillance surveys in Jiangsu province, China, 2016–2021.

**Variable**	**Negative N (%)**	**Recent N (%)**	**OR (95% CI)**	***P*-value**	**aOR (95% CI)**	***P*-value**
**Age group (years)**						
< 25	775 (31.7)	129 (35.0)	1.046 (0.784–1.395)	0.761		
25–39	1,091 (44.6)	148 (40.1)	0.852 (0.645–1.127)	0.262		
≥40	578 (23.6)	92 (24.9)	1.000			
**Marital status with women**						
Married or cohabiting	784 (32.1)	106 (28.7)	1.000			
Single, divorced, or widowed	1,660 (67.9)	263 (71.3)	1.172 (0.921–1.491)	0.197		
**Education level**						
Junior high school or below	634 (25.9)	96 (26.0)	1.000			
Senior high school	755 (30.9)	98 (26.6)	0.857 (0.635–1.158)	0.315		
College or above	1,055 (43.2)	175 (47.4)	1.095 (0.838–1.432)	0.504		
**Registered residence**						
Jiangsu province	1,683 (68.9)	244 (66.1)	1.000			
Other provinces	761 (31.1)	125 (33.9)	1.133 (0.898–1.429)	0.291		
**Duration of living locally**						
< 2 years	837 (34.2)	106 (28.7)	1.000		1.000	
≥2 years	1,607 (65.8)	263 (71.3)	1.292 (1.016–1.644)	0.037	1.366 (1.054–1.770)	0.019
**Recruitment source**						
Venue	783 (32.0)	106 (28.7)	1.000			
Online	831 (34.0)	87 (23.6)	0.773 (0.573–1.044)	0.093		
VCT clinic	830 (34.0)	176 (47.7)	1.566 (1.208–2.031)	0.001		
**Sexual orientation**						
Bisexual or indetermination	879 (36.0)	128 (34.7)	1.000			
Homosexual	1,264 (51.7)	221 (59.9)	1.201 (0.950–1.517)	0.126		
Heterosexual	301 (12.3)	20 (5.4)	0.456 (0.280–0.744)	0.002		
**Main way of finding a sexual partner**						
MSM venue	608 (24.9)	86 (23.3)	1.000			
Internet or dating app	1,836 (75.1)	283 (76.7)	1.090 (0.842–1.411)	0.514		
**Comprehensive HIV knowledge**						
Yes	2,331 (95.4)	338 (91.6)	1.000		1.000	
No	113 (4.6)	31 (8.4)	1.892 (1.251–2.861)	0.003	1.643 (1.046–2.580)	0.031
**UAI in the past 6 months**						
No	2,139 (87.5)	179 (48.5)	1.000		1.000	
Yes	305 (12.5)	190 (51.5)	7.444 (5.875–9.433)	< 0.001	7.373 (5.795–9.380)	< 0.001
**STI in the past 12 months**						
No	2,317 (94.8)	339 (91.9)	1.000			
Yes	127 (5.2)	30 (8.1)	1.615 (1.067–2.442)	0.023		
**HIV testing in the past 12 months**						
Yes	1,242 (50.8)	172 (46.6)	1.000		1.000	
No	1,202 (49.2)	197 (53.4)	1.183 (0.950–1.474)	0.132	1.292 (1.018–1.639)	0.035
**Syphilis infection**						
No	2,309 (94.5)	312 (84.6)	1.000		1.000	
Yes	135 (5.5)	57 (15.4)	3.125 (2.243–4.352)	< 0.001	2.840 (1.972–4.091)	< 0.001

VCT, HIV voluntary counseling and testing; UAI, Unprotected anal intercourse; STI, Sexually transmitted infection.

## Discussion

Although estimates of HIV incidence play a key role in AIDS surveillance, data in China have still been limited in recent years. MSM are a high-risk group for HIV infection in China. HIV prevalence in the 2008–2009 MSM large-scale survey of 61 cities across the country was 4.9% (19). The BED method was used in AIDS sentinel surveillance in Jiangsu province from 2011 to 2015 to estimate that the HIV prevalence among MSM was between 8.1 and 10.6% and the HIV incidence was between 5.1 and 7.8% (5). In this study, the LAg-Avidity-EIA method was applied to estimate that the HIV prevalence was between 8.0 and 9.8% and the HIV incidence was between 5.0 and 9.0% among MSM in Jiangsu province from 2016 to 2021. Compared with the 2011–2015 study, the prevalence was similar, and the estimated incidence could not be simply compared due to different methods used. However, it suggested that HIV incidence among MSM in Jiangsu province still maintained a high level. Compared with HIV incidence among MSM in other countries, the result in our study was higher than that in Manila in the Philippines (20) and lower than those in Mexico (21) and in a study of Myanmar (22). Compared with those in other provinces and cities in China, our result was similar to that of Tang et al.'s study in 20 cities of China (23) and higher than those in Shandong province (24) and Yunnan province (25). The consistently high incidence observed over a 6-year period in our study led us to believe that HIV transmission among MSM in Jiangsu province remains serious and that more effective prevention and control measures need to be implemented.

We observed a significant decrease in the proportion of UAI over the study period, but not in the incidence. This contrast is consistent with the results of other studies (26–28). A possible explanation is that the data on condom use were self-reported. Recall bias cannot be completely avoided. Additionally, participants may provide socially desirable answers to sensitive questions (e.g., condom use) due to the survey mode of face-to-face interview. UAI is still relatively common among MSM; however, promoting condom use alone is not enough to curb the AIDS epidemic among MSM in China. Other innovative and effective HIV prevention strategies should continue to be implemented, including expanding access to HIV testing for key populations, earlier detection of people living with HIV, and more timely prescription of antiretroviral treatment, which are critical to preventing further transmission of HIV (29, 30). In our study, however, MSM who engaged in UAI were more susceptible to HIV infection, which is consistent with findings from many studies (20, 31, 32), which still underscores the importance of promoting condom use. In addition, HIV testing was also an associated factor in our study, and the proportion of MSM tested for HIV in the past year decreased, which may be influenced by the COVID-19 pandemic. At the same time, we should also innovate some new detection modes to increase HIV testing uptake, such as self-testing (33) and transfer testing (self-collecting samples and posting them to the laboratory for testing) (34).

Our study found that education level was not a factor associated with HIV infection among MSM, which may be due to the rapid development of information technology and mobile Internet, making it relatively easy to acquire HIV knowledge. In the past, people with higher education were more likely to access traditional media to acquire knowledge. At present, the influence of educational level on knowledge acquisition is gradually decreasing. However, our study found that MSM with a lack of comprehensive HIV knowledge had a higher risk of HIV infection, which is similar to findings from other studies (35, 36), and health education is still an important strategy for AIDS control and prevention. Consistent with our findings, previous literature suggests that syphilis infection increases the risk of HIV infection (37, 38). Fundamental research has also shown that after syphilis infection, the chance of HIV entering the body through different mechanisms increases (39). The correlation between the two diseases suggests that screening and treatment for sexually transmitted diseases (STDs) are critical to controlling the spread of HIV. STD prevention should be integrated with HIV prevention in policy development.

We selected recently infected MSM as a case group to study the factors associated with HIV infection to reduce the bias caused by changed behaviors of patients with long-term infection, unlike most studies. However, there are some limitations to this study. First, due to the survey mode of face-to-face interview, participants may have provided socially desirable answers to sensitive questions. Interviewers at all survey sites received annual training and followed strict interview protocols to minimize this bias. Second, there might be an overestimation of HIV incidence due to the misclassification of long-term infected persons as recently infected. Excluding unambiguous previously diagnosed cases prior to the LAg-Avidity-EIA testing and adding a calibration factor when estimating the incidence are necessary. Furthermore, we should pay more attention to trends in incidence than the values. Finally, our findings cannot be extrapolated to all MSM in the province and MSM elsewhere.

## Conclusions

This study shows that the incidence of HIV infection among MSM in Jiangsu province remained at a high level from 2016 to 2021. Health education, condom use, and HIV/syphilis testing should continue to be top priorities for HIV prevention among MSM to reduce HIV transmission.

## Data availability statement

The raw data supporting the conclusions of this article will be made available by the authors, without undue reservation.

## Ethics statement

The requirement of ethical approval was waived by Institutional Review Board of Jiangsu Provincial Center for Disease Control and Prevention for the studies involving humans because this study is a routine part of disease control and prevention. The studies were conducted in accordance with the local legislation and institutional requirements. The participants provided their written informed consent to participate in this study.

## Author contributions

YZ: Conceptualization, Methodology, Visualization, Writing—original draft. YC: Investigation, Methodology, Software, Writing—original draft. JL: Data curation, Formal analysis, Investigation, Methodology, Writing—original draft. ZZ: Data curation, Investigation, Methodology, Visualization, Writing—original draft. QS: Investigation, Methodology, Software, Writing—original draft. XL: Project administration, Supervision, Validation, Writing—review & editing. XX: Project administration, Supervision, Validation, Writing—review & editing. XY: Funding acquisition, Methodology, Resources, Writing—review & editing. HH: Conceptualization, Funding acquisition, Methodology, Project administration, Resources, Writing—original draft, Writing—review & editing.
